# Supplementary material for: Seasonal Body Composition Changes in Elite Rugby Players: DXA and Anthropometry-Based Comparison of Backs and Forwards
Source: J Funct Morphol Kinesiol. 2025 Sep 18;10(3):357. doi: 10.3390/jfmk10030357 (PMC12452749; doi:10.3390/jfmk10030357)
Supplement: Supplementary file 1 [file jfmk-10-00357-s001.zip › jfmk-3836862-supplementary.pdf]

Supplementary material

Table S1. Pre-season and end-of-season body composition values measured by DXA and anthropometry.

	Pre-season	End-of- season	*p*-value	Effect size (Cohen´s d)
**DXA**				
TM (kg)	93.25 (15.10)	94.26 (14.99)	0.034[b]	-0.428
LM (kg)	71.71 (8.46)	72.44 (8.12)	0.007[a]	-0.500
FFM (kg)	75.67 (8.98)	76.44 (8.63)	0.005[a]	-0.528
BF (kg)	17.58 (8.07)	17.82 (8.36)	0.625[b]	-0.102
BF (%)	18.98 (5.92)	19.02 (5.98)	0.918[a]	-0.018
BM (kg)	3.96 (0.56)	4.00 (0.56)	0.004[a]	-0.549
BMD (kg/cm2)	1.53 (0.14)	1.53 (0.12)	0.727[a]	-0.062
**Anthropometry**				
Sum6SF (mm)	75.40 (33.52)	77.84 (35.17)	0.246[b]	-0.237
Sum8SF (mm)	100.36 (45.57)	106.52 (50.06)	0.098[b]	-0.337
Endomorphy	2.98 (1.40)	3.06 (1.40)	0.206[b]	-0.276
Mesomorphy	6.68 (1.00)	6.79 (0.98)	0.096[a]	-0.348
Ectomorphy	1.18 (0.70)	1.13 (0.65)	0.217[b]	0.319

Data are expressed as mean (standard deviation). [a]Paired t-test, [b]Wilcoxon sign rank test. Abbreviatures: BF (body fat), BM (bone mass), BMC (bone mass content), BMD (bone mineral density), FFM (fat-free mass), LM (lean mass), TM (total mass), Sum 6SF (sum of six site skinfolds), Sum 8SF (sum of eight site skinfolds).

Table S2. Pre-season and end-of-season DXA and anthropometric body composition values in relation to position (front rows and other forwards) and differences in each position throughout the season.

Body composition variables	Moment of the season	FR (n = 8)			OF (n=8)		
		Mean (SD)	p-value (intra-group)	Effect size (Cohen´s d)	Mean (SD)	p-value (intra-group)	Effect size (Cohen´s d)
**DXA**							
TM (kg)	PS	110.350 (8.975)	0.382[a]	-0.330	101.793 (7.510)	0.750[a]	0.117
	ES	112.161 (10.972)			101.454 (4.998)*		
LM (kg)	PS	77.322 (6.681)	0.037[a]	-0.906	77.411 (3.214)	0.155[a]	0.563
	ES	78.584 (7.435)			78.174 (4.847)		
FFM (kg)	PS	81.614 (7.225)	0.032[a]	-0.945	83.135 (5.583)	0.186[a]	0.519
	ES	82.915 (7.987)			82.623 (5.053)		
BF (kg)	PS	28.736 (5.794)	0.784[a]	-0.100	18.658 (4.235)*	0.853[a]	-0.068
	ES	29.246 (7.566)			18.832 (2.578)*		
BF (%)	PS	27.008 (4.562)	0.940[a]	0.028	19.062 (3.452)*	0.633[a]	-0.176
	ES	26.910 (5.582)			18.832 (2.578)*		
BM (kg)	PS	4.291 (0.631)	0.078[a]	-0.330	4.422 (0.260)	0.251[a]	0.117
	ES	4.331 (0.618)			4.449 (0.242)		
BMD (kg/cm^2)	PS	1.608 (0.146)	0.979[a]	0.010	1.631 (0.074)	0.494[a]	0.255
	ES	1.607 (0.137)			1.616 (0.047)		
**Anthropometry**							
Sum6SF (mm)	PS	123.075 (23.647)	0.579[a]	-0.206	75.250 (19.732)*	0.623[a]	-0.182
	ES	128.925 (26.112)			77.088 (13.503)*		
Sum8SF (mm)	PS	162.838 (34.291)	0.356[a]	-0.349	102.287 (30.292)*	0.807[a]	-0.089
	ES	179.500 (37.796)			103.725 (20.490)*		
Endomorphy	PS	5.063 (0.927)	0.792[a]	-0.097	2.725 (0.717)*	0.514[a]	-0.243

	ES	128.925 (26.112)			2.813 (0.533)*		
Mesomorphy	PS	7.787 (0.479)	0.400[a]	-0.317	7.175 (0.725)*	0.317[a]	0.381
	ES	7.925 (0.614)			7.088 (0.736)*		
Ectomorphy	PS	0.175 (0.175)	1.000[b]	-0.317	1.125 (0.282)*	0.388[a]	-0.325
	ES	0.200 (0.185)			1.188 (0.304)*		

Data are expressed as mean (standard deviation). Abbreviatures: BF (body fat), BM (bone mass), ES (end-of-season), FFM (fat-free mass), LM (lean mass), PS (pre-season), TM (total mass), Sum 6SF (sum of six site skinfolds), Sum 8SF (sum of eight site skinfolds). *Significant differences between front rows and other forwards. [a] Paired t-test, [b] Wilcoxon sign rank test.
